# Comparative models on low multiplier DRG classification for advanced lung cancer

**DOI:** 10.3389/fpubh.2025.1614938

**Published:** 2025-09-12

**Authors:** Mingming Yu, Yanxi Zhang

**Affiliations:** ^1^School of Economics and Management, Shanghai Technical Institute of Electronics & Information, Shanghai, China; ^2^School of Economics and Management, Tongji Zhejiang College, Jiaxing, China; ^3^Institute of Medical Engineering and Translational Medicine, Tianjin University, Tianjin, China; ^4^Department of Medical Insurance Administration, The First Affiliated Hospital, College of Medicine, Zhejiang University, Hangzhou, China

**Keywords:** machine learning, advanced lung cancer, low multiplier DRGs, prediction model, upcoding

## Abstract

**Objective:**

This study aimed to compare the performance of machine learning models in predicting low multiplier DRGs for advanced lung cancer, and to identify the optimal algorithm along with key influencing factors.

**Methods:**

Prediction models for low multiplier DRGs in advanced lung cancer were developed using four machine learning algorithms: logistic regression, hybrid naive Bayes, support vector machine (SVM), and random forest. Model performance was evaluated, and key contributing features were identified.

**Results:**

The random forest algorithm achieved the highest AUC, accuracy, and precision across all three ER group, indicating robust performance. Second, cost-related features and length of hospital stay (LoS) reflecting “resource consumption” contributed significantly more to the low multiplier DRGs prediction than demographic factors such as gender and age.

**Conclusion:**

Based on comorbidity severity, the DRG classification for advanced lung cancer patients receiving internal medicine treatment under ER1 appeared reasonably structured and provided a valid basis for subgroup comparisons. Additionally, according to the predictive model’s findings, potential signs of upcoding and intentional underuse of reimbursable medications were observed, highlighting the need to monitor examination fee reductions across ER1 subgroups and to track medication costs in ER11 throughout the hospital stay. Lastly, in predicting low multiplier DRGs, larger datasets improve model stability. Model choice should align with the analytical goal: Random Forest offers higher precision and robustness, while logistic regression or SVM may be preferred for higher recall.

## 1 Introduction

The DRG-based medical insurance payment system has increasingly been adopted worldwide to address rising hospital costs, rather than relying on cost-based payments (1–3). At its core, the DRG payment method introduces the concept of social average cost, calculated from large-scale historical healthcare data (4). Under this system, patients classified in the same diagnosis group are reimbursed based on the average treatment cost across all medical institutions within a specific region or district. Low multiplier cases refer to those with medical expenses lower than the average cost of a given DRG group. If the DRG payment amount remains fixed, hospitals may profit from such cases. In contrast, high multiplier cases have medical expenses exceeding the average cost of the DRG group. If payments continue to be based on the standard DRG amount, hospitals or physicians may have to bear financial losses (5).

DRGs and their associated bundled metrics, such as high and low multiplier cases and the case-mix index, play a central role in hospital operations and financial performance. Therefore, accurate grouping is essential. According to the U.S. MS-DRG and China’s CHS-DRG coding guidelines, the core principles guiding DRG categorization include: cases with varying disease types should be classified separately; patients diagnosed with the same condition but undergoing different treatments should be assigned to distinct groups; and even among patients with identical diagnoses and treatment approaches, individual attributes—such as age, gender, and the presence of comorbidities or complications—necessitate further subdivision (6, 7).

When DRGs are accurately classified, it becomes easier to identify cases of high or low multiplier. Traditionally, the assignment of DRGs has been a labor-intensive manual process, typically performed by coding specialists after a patient’s discharge. For financial gain, hospitals may sometimes engage in fraudulent practices by intentionally upcoding DRG assignments (8), which can lead to low multiplier cases, as the inflated DRG weight does not match actual treatment costs. The phenomenon of upcoding has been reported in many countries (8–10). A RAND review summarized the key characteristics of studies on upcoding practices in provider settings. Most of the included articles were published after 2015 (62%), and the majority were conducted in the United States (59%). Of the 13 studies that investigated upcoding for individual admissions or visits, most did so by validating the accuracy of a coding intensity measure compared with specific indicators of severity of a patient’s admission, usually from administrative claims data (11). Apart from upcoding, even when accurately assigned to a DRG group, hospitals or doctors may still seek to maximize profits by intentionally withholding necessary treatments (12). Both of these practices can contribute to the emergence of low multiplier DRG cases (12). Mostly, when regulatory authorities or payers attempt to monitor such behaviors, they often rely on manual sampling methods. However, this manual inspection method is time-consuming, labor-intensive, and significantly influenced by the subjective judgments and random errors of the inspectors, making proactive control challenging. A U.S. study suggested future work should investigate further the potential role of technological change in estimating the extent of upcoding (13). It is precisely for the above purpose; this study tried to use machine learning to distinguish between normal and abnormal medical behaviors based on key factors or characteristics affecting DRG low-multiplicity.

Machine learning techniques have recently been applied to a wide range of healthcare applications (14). In healthcare, machine learning has been employed to address classification tasks, develop predictive models, and identify high-risk patients. In both academic and applied researches, the automatic grouping or coding of DRGs—i.e., assigning cases to appropriate DRG categories based on medical records—has become the mainstream research focus. For example, a study proposed a data-driven grouping approach optimized through machine learning algorithms, demonstrating that, when appropriate algorithms are selected, data-based grouping can achieve classification performance comparable to traditional expert-defined grouping methods (14). Furthermore, Wang et al. introduced DRG-LLaMA, a state-of-the-art large language model (LLM) fine-tuned on clinical notes to improve DRG assignment. Their results indicated that DRG-LLaMA outperformed previously established models in DRG prediction accuracy (15).

In contrast, studies related to DRG-based cost prediction and automatic cost classification remain relatively limited. This is largely due to the sensitive nature of cost data, which makes it difficult to access, as well as the complexity of factors influencing healthcare costs. On the contrary, automatic DRG grouping is more feasible because it typically relies on structured data from the discharge summary. Nonetheless, some key studies on automatic cost prediction based on DRGs have begun to emerge. Studies have leveraged deep learning and natural language processing (NLP) models to improve early cost estimation accuracy. For instance, Liu et al. employed NLP models to predict DRGs and the corresponding case mix index (CMI) using clinical notes and structured ICU data, in order to estimate hospital costs in an acute care setting. Their method demonstrated high predictive accuracy, with an absolute CMI error of less than 2.5% (16). In addition, many researchers applied machine learning algorithms such as random forests, support vector machines and neural network to predict medical expenses, with random forests yielding the highest accuracy (17–19).

Due to the numerous DRG disease groups for various conditions, this study focused solely on advanced primary lung cancer. Studies from many countries showed that most newly diagnosed lung cancer cases are at an advanced stage, including stage III and IV disease, most of which is inoperable and can only be treated with medical or radiation therapy (20, 21). The most common type of lung cancer is non-small cell lung cancer (NSCLC) (20). In recent years, the treatment landscape for NSCLC has undergone a paradigm shift from chemotherapy to targeted therapies and immune checkpoint inhibitors (ICIs) (22). In the U. S., the total costs of NSCLC have been increasing, mainly driven by outpatient costs for systemic therapy, which might reflect the greater use of ICIs for advanced NSCLC (22). A study from Mexico found that patients with stage IV NSCLC showed considerable variation in active treatment regimens (21). In addition, data from China showed that among 174 primary lung cancer patients admitted to a hospital in 2019, medication use was assessed, revealing that an unreasonably high proportion—83.9%—of treatment plans were considered inappropriate. Only 28 medical records, or 16.1%, had rational anti-cancer drug treatment plans. Furthermore, the incidence of adverse drug reactions (ADRs) was 32.2%, with chemotherapy-induced ADRs occurring in 44.4% of cases and targeted therapy-induced ADRs occurring in 7.14% (23). In recent years, with the increasing variability and complexity in the treatment of advanced non-surgical lung cancer, concerns have emerged that hospitals may intentionally reduce inpatient costs to avoid losses under the DRG payment system. Meanwhile, they may also increase unnecessary treatments requiring out-of-pocket payments, thereby adding to patients’ financial burden (12). These behaviors may ultimately lead to low multiplier DRGs.

In China, according to version 1.0 of the CHS-DRG, patients with primary lung cancer receiving internal medicine treatment are classified under Major Diagnostic Category (MDC) E (Respiratory System Diseases and Disorders). The core disease-related grouping (ADRG) codes for this category include ER11, ER13, and ER15, where “R” denotes the internal medicine section. ER11 refers to respiratory system neoplasms with major complications or comorbidities, ER13 refers to moderate cases with some complications or comorbidities, and ER15 refers to cases without major complications or comorbidities. Additionally, this grouping encompasses diagnoses coded as C33-C34, representing different locations of malignant lung tumors, along with other diagnoses indicating (severe) complications or comorbidities, and specific treatments such as targeted therapy and palliative care (7). However, this classification primarily reflects the “clinical process” aspect of DRG grouping and does not adequately address “resource consumption” (e.g., medications, supplies, length of stay, readmission rates) or “patient characteristics” (e.g., age, gender) (7).

First, this study aimed to utilize the CHS-DRG grouping scheme to qualitatively classify advanced primary lung cancer cases treated with internal medicine. Subsequently, machine learning classification models were developed to predict DRG multiplier associated with the average cost of a given DRG group. By analyzing sample data, the study sought to identify potential relationships between patient characteristics and DRG multiplier, providing a quantitative basis for scientific DRG supervision. Four machine learning models—logistic regression, naive bayes, support vector machine, and random forest—were employed for classification. The performance of these models was evaluated and compared based on accuracy, sensitivity, specificity, and the area under the receiver operating characteristic (ROC) curve. The objective was to determine the most suitable model for predicting DRG multiplier within DRG groups related to advanced primary lung cancer. Ultimately, the study aimed to facilitate intelligent monitoring of upcoding and inappropriate treatments, particularly the under-provision of care within insurance-covered services, thereby addressing issues associated with low multiplier DRGs.

## 2 Construction of machine learning models

### 2.1 Algorithm descriptions

In this study, four machine learning models were selected for analysis:

1. Random forest algorithm

Random forest, introduced by Breiman (24), is an ensemble learning method that builds a collection of decision trees using random subsets of features and training data (bagging). Each tree makes a prediction, and the final classification result is determined by majority voting. This approach reduces overfitting risk and improves generalization.
Random forest is particularly effective in handling structured data with complex, nonlinear interactions among features. It is robust to outliers, can model feature importance, and performs well even without extensive feature engineering. Random forest has consistently demonstrated strong predictive performance in numerous studies related to medical cost estimation (17–19).

2. Naive bayes algorithm

The naive bayes classifier is a probabilistic model based on Bayes’ theorem, which assumes conditional independence among features given the class label. The advantage of Gaussian Naïve Bayes probability prediction is that, it is computationally efficient and can handle large data sets with high dimensionality. Data of mixed data values is also handled efficiently (25). Clinically, Naïve Bayes aids in disease prediction by evaluating the likelihood of diagnoses based on symptoms, test results, or patient demographics, using the probability of each category to make a classification. Additionally, the probabilistic outputs of Naïve Bayes provide valuable prediction confidence levels, supporting critical decision-making in healthcare (26).
In this study, we adopted a hybrid naive bayes algorithm, which integrates different modeling strategies for continuous and categorical variables. Continuous variables, such as total cost or LoS, are assumed to follow a Gaussian distribution and are modeled using the Gaussian Naive Bayes subcomponent. This involves estimating the mean and standard deviation for each class, and computing likelihoods under the normal distribution assumption. On the other hand, categorical variables, such as gender or ICD codes, are treated as discrete features and modeled using the Categorical Naive Bayes framework, depending on the encoding strategy. This hybrid approach preserves the original information without discretization and is well-suited to heterogeneous medical cost dataset.

3. Support vector machine (SVM) algorithm

The Support Vector Machine (SVM), proposed by Cortes and Vapnik in 1995 (27), is a powerful classification technique that seeks to find the optimal hyperplane that separates classes in the feature space. By using kernel functions, SVMs can handle both linear and nonlinear decision boundaries, making them highly adaptable.
  SVMs are considered highly effective when combined with Principal Component Analysis (PCA) for feature reduction. And SVMs are well-suited for classification tasks with clear class boundaries and relatively few outliers. For instance, Kuo et al. employed SVMs to predict mortality rates among hospitalized motorcycle riders (28).

4. Logistic regression algorithm

Logistic regression is a widely used classification model that estimates the probability of a binary outcome based on one or more input features. It models the log-odds of the response variable as a linear combination of the input features and employs the logistic (sigmoid) function to constrain output values between 0 and 1.
This algorithm is especially appropriate for structured and interpretable classification tasks, particularly when the input features have a roughly linear relationship with the log-odds of the target. In medical research, logistic regression is extensively used to predict disease occurrence, treatment outcomes, and survival probabilities (29). The above four machine learning classifiers were implemented and compared within the Python 3.13.1 environment.

### 2.2 Data source and preprocessing

This study utilized data from the medical insurance management system of a tertiary general hospital in Zhejiang Province. A total of 12,640 inpatient cases of internal medicine hospitalizations for lung cancer were collected between January 1, 2022, and December 31, 2024. The collected data included patient demographics (age and gender), primary diagnosis and corresponding ICD codes, length of hospital stay (LoS), and detailed hospitalization costs.

According to the CHS-DRG, the dataset was curated and subjected to the following preprocessing steps to yield a final cohort of 2,324 cases: including 631 cases of ER11 (respiratory system tumor with serious complications or comorbidities), 1,305 cases of ER13 (respiratory system tumor with general complications or comorbidities), and 388 cases of ER15 (respiratory system tumor without complications or comorbidities).

1. Duplicate removal: eliminated redundant records from the dataset.
2. Handling missing data: retrieved missing information from patient records where possible; cases with unresolvable missing data were excluded.
3. Adjustment for medical price index: normalized all cost data from 2022 to 2024 to the 2024 baseline, accounting for medical price inflation.
4. Exclusion of self-pay cases: removed cases involving self-paying patients, focusing the study on DRG cases covered by medical insurance.

### 2.3 Descriptive statistical analysis

Data were analyzed using Python version 3.13.1. Categorical variables were summarized using frequencies and percentages, while continuous variables were described using median, IQR/Median and Skewness. Differences in medication costs, material costs, blood costs, inspection and examination fees, and LoS among the ER11, ER13, and ER15 groups were assessed using Kruskal-Wallis H test. A *p*-value of less than 0.05 was considered statistically significant.

Table 1 presented the results, indicating that the proportion of male patients was significantly higher than that of female patients across the ER11, ER13, and ER15 groups. This finding aligned with data from the National Cancer Center’s “China Cancer Statistics Report,” which reported that, as of July 2024, approximately 70–75% of lung cancer patients in China were male, and 25–30% were female (30). The gender distribution in our study’s ER groups was similar, with a slightly higher proportion of females in the ER15 group.

**Table 1:** Frequency and Proportion of Categorical Variables Among ER Patient Groups.

Variable	Category	ER11 Frequency	Proportion of ER11	ER13 Frequency	Proportion of ER13	ER15 Frequency	Proportion of ER15
Gender	Male	491	77.8%	936	71.7%	245	63.1%
	Female	140	22.2%	369	28.3%	143	36.9%
Age	<60 years	130	20.6%	272	20.8%	131	33.8%
	60–74 years	162	25.7%	304	23.3%	195	50.3%
	≥75 years	339	53.7%	729	55.9%	62	15.9%
ICD Primary Diagnosis Code	C34.900×001	385	61.0%	814	62.4%	219	56.4%
	C34.900×004	101	16.1%	226	17.3%	74	19.1%
	C34.900×005	141	22.3%	264	20.3%	95	24.5%
	C34.900×006	4	0.6%	1	0	0	0
DRG Multipliers	High	48	7.6%	24	1.8%	29	7.4%	Low	103	16.3%	246	18.9%	78	20.2%	Normal	480	76.1%	1,035	79.3%	281	72.4%

The aforementioned report also indicated that individuals aged 50–70 constitute approximately 60–70% of lung cancer cases, while those under 40 account for about 5–10% (30). See Table 1 in our study, cases under 50 years old were rare, and the number of cases under 60 was lower than those aged 60 to 74. Therefore, we categorized patients accordingly. Post-categorization, it was observed that in the ER11 and ER13 groups, patient numbers increased with age, with those aged 75 and above comprising over half of the cases, and the ER15 group had a smaller proportion of patients aged 75 and above (15.9%). This discrepancy between the report and our study may stem from regional differences; the national report reflects data across China, whereas our study focuses on Zhejiang Province. Notably, in 2019, Zhejiang’s average life expectancy was 79.1 years, among the highest nationwide (31). Consequently, the higher proportion of patients aged 75 and above in the ER11 and ER13 groups may be attributable to this increased longevity. Additionally, the higher proportion of patients aged 75 and above in the ER11 and ER13 groups may be explained by the greater prevalence of comorbidities and complications in this age group.

In our study, among the three groups of ER cases, the proportion of patients with ICD main diagnostic code C34.900×001 (lung malignant tumor) exceeded 50%, while the proportion of patients with C34.900×006 (bilateral lung malignant tumor) was very small (Table 1), and the proportions of patients diagnosed with C34.900×004 (malignant neoplasm of the left lung) and C34.900×005 (malignant neoplasm of the right lung) were moderate. As also shown in Table 1, the proportion of high multiplier DRG cases remained consistently low across all three groups—7.6% in ER11 (48 cases), 1.8% in ER13 (24 cases), and 7.4% in ER15 (29 cases).

**Figure 1:**
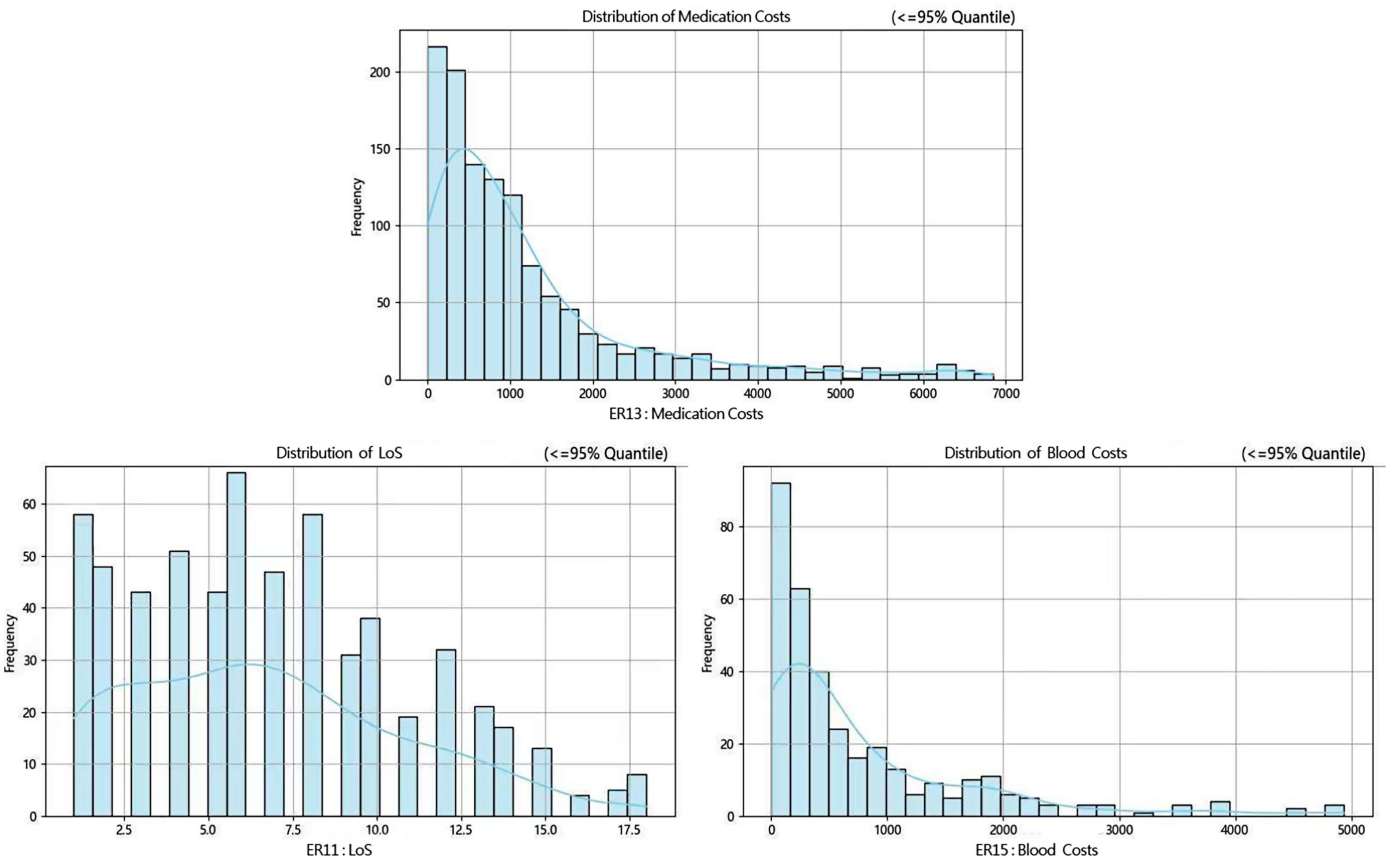
Probability distribution plots of selected costs and LoS in ER11, ER13 and ER15.

Then, according to many studies, inpatient cost data mostly exhibit a right-skewed distribution (32, 33). Therefore, we conducted a skewness analysis on the distributions of various cost-related variables and LoS in ER11, ER13, and ER15. All of these variables demonstrated significant right-skewness. A skewness value greater than +1 was considered to indicate significant right-skewness (Tables 2–6). Also, the Figure 1 below presents a visualization analysis of the probability distributions for selected data from the three groups as examples.

**Table 2:** Median, IQR/Median, and Skewness of medication costs among ER patient groups.

Groups	Number of cases	Median	IQR / Median	Skewness	*H*	*p*
ER11	631	2454.2	1.9	9.9	338.329	<0.001
ER13	1,305	822.7	1.7	7.2		
ER15	388	439.4	2.7	4.2		

**Table 3:** Median, IQR/Median, and Skewness of material costs among ER patient groups.

Groups	Number of cases	Median	IQR/Median	Skewness	*H*	*p*
ER11	631	691.9	1.3	6.9	43.930	<0.001
ER13	1,305	516.1	1.0	6.5		
ER15	388	440.4	1.1	3.9		

**Table 4:** Median, IQR/Median, and Skewness of blood costs among ER patient groups.

Groups	Number of cases	Median	IQR/Median	Skewness	*H*	*p*
ER11	631	2454.7	2.0	9.9	343.180	<0.001
ER13	1,305	782.5	1.7	7.6		
ER15	388	438.4	2.5	4.4		

**Table 5:** Median, IQR/Median, and Skewness of inspection and examination fees among ER patient groups.

Groups	Number of cases	Median	IQR/Median	Skewness	*H*	*p*
ER11	631	6990.4	0.8	3.1	136.889	<0.001
ER13	1,305	5498.4	0.8	1.7		
ER15	388	3985.4	0.7	1.6		

**Table 6:** Median, IQR/Median, and Skewness of length of stay (LoS) among ER patient groups.

Groups	Number of cases	Median	IQR/Median	Skewness	*H*	*p*
ER11	631	6.0	1.0	10.5	306.305	<0.001
ER13	1,305	4.0	1.0	2.9		
ER15	388	2.0	1.5	2.7		

Because the data are right-skewed, this study used the more robust variability measure, IQR/Median, to assess relative dispersion. The median and IQR/Median for medication costs, material costs, blood costs, inspection and examination fees, and LoS across the ER11, ER13, and ER15 groups are presented in the below tables. An IQR/Median value greater than 1.0 indicates that the data are highly dispersed, with a large relative variability; a value between 0.3 and 1.0 suggests moderate dispersion, with a medium level of relative variability. Notably, each ER group, the IQR/Median values for medication costs, material costs, blood costs, and LoS exceed or equal 1, indicating high dispersion and significant individual variability in these expenses. In contrast, only the IQR/Median values for inspection and examination fees are below 1, suggesting greater consistency within the indicator. Subsequent the Kruskal-Wallis H test revealed statistically significant differences among the ER groups in terms of medication costs, material costs, blood costs, inspection and examination fees, and LoS (*p* < 0.001). Further pairwise comparisons using Dunn’s *post hoc* test revealed that, except for the difference in material costs between groups ER13 and ER15, which was not statistically significant (*p* = 0.483), all other indicators showed significant differences among the three ER groups (*p* < 0.001). These findings underscore the effectiveness of the DRG classification system (ER11, 13, 15) in differentiating patient groups (Tables 2–6).

### 2.4 Construction and results of predictive models

This study utilized data from patient groups ER11, ER13, and ER15, incorporating categorical variables (age, gender, primary ICD diagnosis and corresponding codes) and continuous variables (medication costs, material costs, blood costs, inspection and examination fees, and length of hospital stay) as feature variables. The DRG cases with low or normal multiplicity served as the target variable. Originally, the total sample sizes of the three groups—ER11, ER13, and ER15—were 631, 1,305, and 388, respectively. After excluding the high multiplier DRG cases, the remaining samples—including only normal and low multiplier DRGs—were reduced to 583, 1,281, and 359, respectively.

Four machine learning algorithms were employed to develop models capable of predicting DRG cases. The performance of these algorithms in predicting DRG cases was compared to identify the optimal predictive model and to explore factors influencing the target variable. The goal was to provide decision support for early intervention and prevention of abnormal medical processes.

#### 2.4.1 Data and model preparation

Initially, since many feature variables—especially cost-related ones—exhibit clear right-skewed distributions, such skewness may affect machine learning model performance by distorting feature weight learning and other behaviors. Therefore, we first identified all skewed features, and applied a logarithmic transformation using NumPy’s np.log1p function to those with a skewness greater than ±1 to make their distributions more normal-like. After that, the feature variables underwent normalization, scaling the data to a [0, 1] range. Subsequently, data from the ER11, ER13, and ER15 groups were randomly shuffled and partitioned. The experimental method used in this study was 5-fold cross-validation, more precisely, stratified 5-fold cross-validation. The dataset was only split into training and validation sets, without a separate test set. This decision was made for several reasons: the three groups in the dataset contain relatively limited samples—583, 1,281, and 359 cases, respectively. Further splitting out a test set would have significantly affected the representativeness of the data. Moreover, the purpose of this study is a comparative analysis of different models, rather than the deployment of a final predictive model. Therefore, omitting a test set is acceptable under such research goals, as supported by relevant literature (34, 35).

While using a separate test set is indeed helpful for evaluating a model’s generalization ability on unseen data, our cross-validation design took a different approach. To reduce the risk of overfitting and prevent potential validation leakage during cross-validation, we chose to use fixed (default) hyperparameters instead of performing grid search over a predefined set of values. This approach ensures that no fold is indirectly optimized during the tuning process, thus providing a more reliable estimate of model generalization performance.

In our implementation, several key hyperparameters were either explicitly specified or used with their default values to ensure reproducibility and robustness. For the Random Forest classifier, we set random_state = 42 to ensure reproducibility. The number of trees (n_estimators) was left at the default value of 100.

For the naive bayes classifier, model evaluation was performed using a fixed random seed (random_state = 42). For classification, we applied a hybrid naive bayes approach using GaussianNB and CategoricalNB, both with default hyperparameters, including variance smoothing (1e-9) and Laplace smoothing (alpha = 1.0), respectively.

For the logistic regression classifier, the model is configured with random_state = 42 and max_iter = 500, while other hyperparameters such as penalty, solver, and C remain at their default settings.

For the support vector machine (SVM) classifier, the model is an SVM (SVC) with kernel = “rbf” and random_state = 42.

Additionally, an analysis of the class label distributions in the ER11, ER13, and ER15 groups revealed that the ratios of normal to low DRG multipliers were approximately 4.7:1, 4.2:1, and 3.6:1, respectively. To mitigate the impact of class imbalance, the stratified k-fold cross-validation and the compute_sample_weight function from Python’s scikit-learn library was utilized. The core idea of the stratified k-fold cross-validation is to ensure that the proportion of each class in every fold remains consistent with the overall class distribution of the dataset during the splitting process. And the compute_sample_weight function calculates sample weights inversely proportional to class frequencies, assigning higher weights to minority class samples, thereby enabling the model to focus more on these underrepresented cases.

#### 2.4.2 Model evaluation metrics

The influencing factors for predicting the DRG multiplier were identified based on the importance rankings of various indicators. In 5-fold cross-validation, the data was divided into 5 subsets (folds). The models were trained on four subsets and validated on the remaining one. This process was repeated five times. For each iteration, a set of evaluation metrics—including Accuracy, Precision, Recall, F1-score, and AUC (Area Under the ROC Curve)—was computed. The final performance evaluation was obtained by averaging each of these metrics across the five folds. Corresponding standard deviations were also calculated to reflect the stability of model performance across folds. Specifically: the accuracy measures the models’ overall ability to correctly classify both ‘low multiplier’ and ‘normal multiplier’ cases; The precision indicates the proportion of cases predicted as ‘low multiplier’ that are indeed ‘low multiplier’ cases; The recall (sensitivity) reflects the proportion of actual ‘low multiplier’ cases that were correctly identified by the models. AUC represents the area under the ROC curve, illustrating the trade-off between sensitivity (recall) and the false positive rate, a higher AUC indicates better model performance.

The commonly used evaluation metrics, along with their formulas and explanations, are presented below (Equation 1).


(1) $$\text{Accuracy}=\frac{\mathrm{TP}+\mathrm{TN}}{\mathrm{TP}+\mathrm{TN}+\mathrm{FP}+\mathrm{FN}}$$

Accuracy is the most intuitive classification metric. It represents the proportion of correctly classified samples out of the total number of samples (Equation 2).


(2) $$\text{Precision}=\frac{\mathrm{TP}}{\mathrm{TP}+\mathrm{FP}}$$

Precision refers to the proportion of true positive samples among all samples that are predicted as positive. A higher precision indicates that the model is more accurate in identifying positive cases (Equation 3).


(3) $$\text{Recall}=\frac{\mathrm{TP}}{\mathrm{TP}+\mathrm{FN}}$$

Recall (also known as sensitivity) is the proportion of actual positive samples that are correctly identified by the model. A higher recall means the model can detect more of the actual positive cases (Equation 4).


(4) $$F1-\text{score}=2\times \frac{\text{Precision}\times \text{Recall}}{\text{Precision}+\text{Recall}}$$

F1-score is the harmonic mean of precision and recall. It balances both precision and recall, making it especially useful when there is an imbalance between them. A higher F1-score indicates better overall performance of the model.

The components of the confusion matrix are defined as follows: TP (True Positive) refers to the number of positive samples correctly predicted as positive; TN (True Negative) is the number of negative samples correctly predicted as negative; FP (False Positive) denotes the number of negative samples incorrectly predicted as positive; and FN (False Negative) represents the number of positive samples incorrectly predicted as negative.

#### 2.4.3 Comparison of predictive model performance

**Figure 2:**
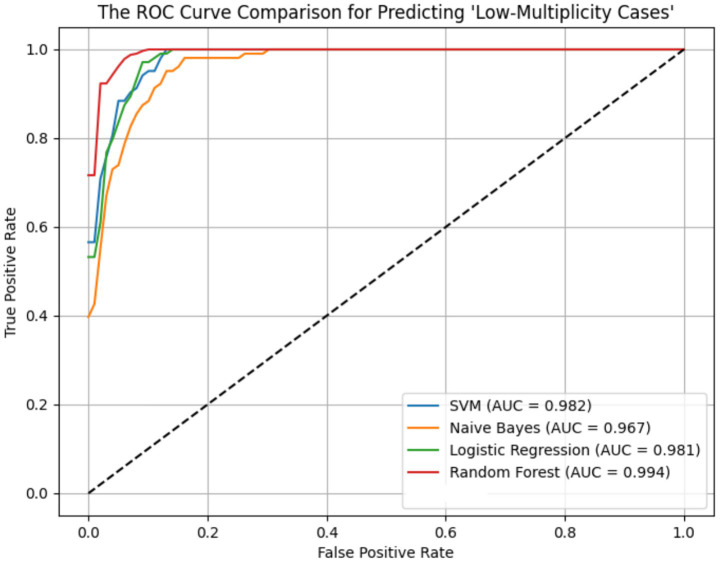
ROC curve of the prediction models for ‘low-multiplicity cases’ in the ER11 group.

**Figure 3:**
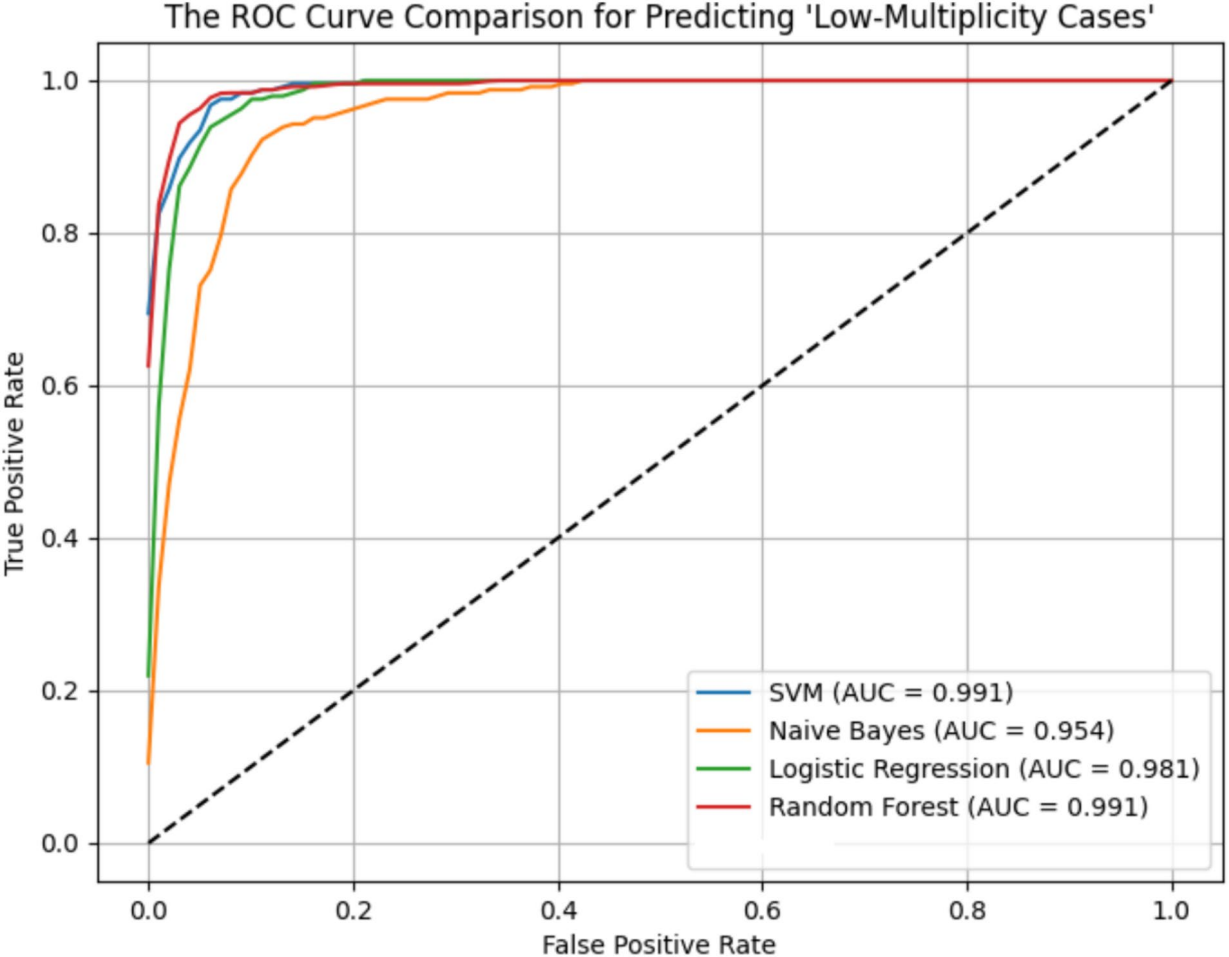
ROC curve of the prediction models for ‘low-multiplicity cases’ in the ER13 group.

**Figure 4:**
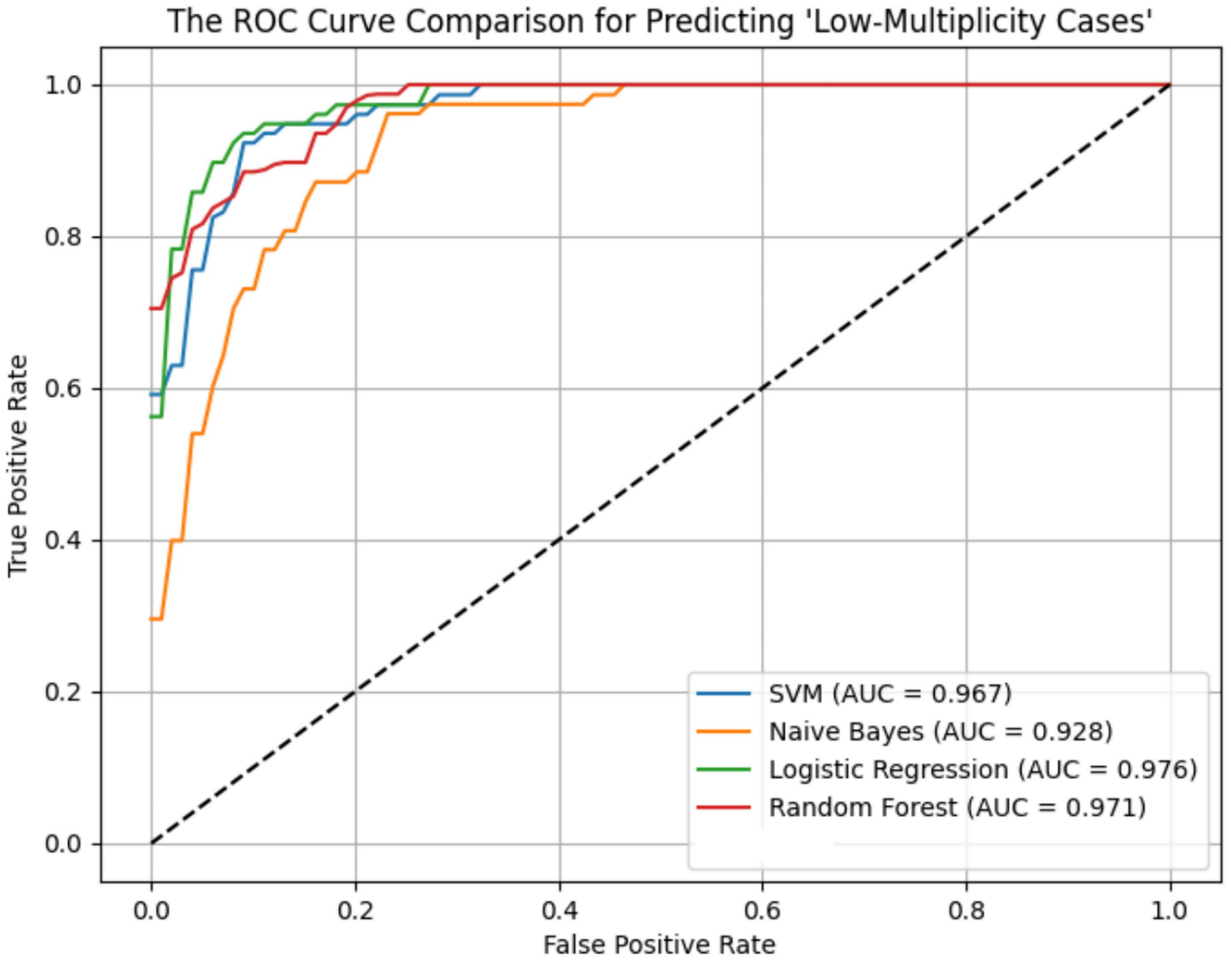
ROC curve of the prediction models for ‘low-multiplicity cases’ in the ER15 group.

ROC curves for the four models across the ER11, ER13, and ER15 groups were presented in Figures 2–4. The results were largely consistent across these groups. The areas under the ROC curves of the three hybrid bayesian models were the smallest, indicating the worst predictive performance. Except for a slightly lower performance in the ER15 group, the random forest model outperformed both the logistic regression and support vector machine models, indicating superior predictive performance. In addition, it can be seen that the ROC curves of the ER13 group were the smoothest, while the ER15 group were the most curved. ER13 had the largest sample size while ER15 had the smallest, indicating that the larger the sample size, the better the model generalization, and the more stable the prediction. In general, all four models performed well across the three ER groups, with AUCs greater than 0.9.

Additionally, as shown in Tables 7–9, the random forest model performed best in terms of accuracy, precision and AUC, making it suitable for precise prediction of low-multiplicity cases. However, it should be noted that the recall varied substantially, with somewhat high standard deviations. Logistic regression and support vector machine models achieved higher recall, enabling better coverage of low-multiplicity cases and making them suitable for scenarios where minimizing missed diagnoses is critical, though at the cost of slightly lower precision. The overall performance of the naive bayes model was relatively weaker, particularly in terms of precision, suggesting it may not be suitable for use on its own for this task.

**Table 7:** Performance evaluation metrics of prediction models for low multiplier cases in the ER11 group.

Model	Accuracy (standard deviation)	Precision (standard deviation)	Recall (standard deviation)	F1 Score (standard deviation)	AUC (standard deviation)
Hybrid Bayesian	0.8507 (0.0203)	0.7528 (0.0816)	0.8352 (0.0712)	0.7898 (0.0642)	0.9673 (0.0121)
Random Forest	0.9314 (0.0093)	0.9249 (0.0253)	0.8357 (0.0647)	0.8770 (0.0417)	0.9939 (0.0029)
Logistic Regression	0.8971 (0.0123)	0.7589 (0.0370)	0.9419 (0.0367)	0.8403 (0.0350)	0.9807 (0.0089)
SVM	0.8936 (0.0318)	0.7187 (0.0631)	0.9514 (0.0437)	0.8183 (0.0545)	0.9817 (0.0103)

**Table 8:** Performance evaluation metrics of prediction models for low multiplier cases in the ER13 group.

Model	Accuracy (standard deviation)	Precision (standard deviation)	Recall (standard deviation)	F1 Score (standard deviation)	AUC (standard deviation)
Hybrid Bayesian	0.8915 (0.0211)	0.6576 (0.0476)	0.9227 (0.0240)	0.7668 (0.0349)	0.9542 (0.0152)
Random Forest	0.9547 (0.0153)	0.9050 (0.0293)	0.8540 (0.0623)	0.8780 (0.0429)	0.9907 (0.0046)
Logistic Regression	0.9290 (0.0197)	0.7470 (0.0503)	0.9593 (0.0224)	0.8394 (0.0393)	0.9814 (0.0075)
SVM	0.9399 (0.0112)	0.7839 (0.0327)	0.9513 (0.0276)	0.8590 (0.0239)	0.9910 (0.0047)

**Table 9:** Performance evaluation metrics of prediction models for low multiplier cases in the ER15 group.

Model	Accuracy (standard deviation)	Precision (standard deviation)	Recall (standard deviation)	F1 Score (standard deviation)	AUC (standard deviation)
Hybrid Bayesian	0.8441 (0.0317)	0.6097 (0.0560)	0.8450 (0.0884)	0.7034 (0.0388)	0.9283 (0.0228)
Random Forest	0.9248 (0.0206)	0.9072 (0.0517)	0.7292 (0.1012)	0.8039 (0.0717)	0.9710 (0.0192)
Logistic Regression	0.9110 (0.0356)	0.7591 (0.1052)	0.9083 (0.0687)	0.8198 (0.0550)	0.9757 (0.0102)
SVM	0.8887 (0.0289)	0.6791 (0.0511)	0.9350 (0.0578)	0.7859 (0.0482)	0.9670 (0.0167)

From the perspective of variance, the random forest model exhibited the smallest overall standard deviation, with virtually no fluctuation in AUC, making it the most stable and robust model, suitable for generalization. In contrast, the naive bayes models showed the largest variability, with relatively high variance across all metrics, indicating the weakest robustness. Logistic regression and support vector machines fell in between, demonstrating moderate stability.

Among the three datasets—ER11, ER13, and ER15—the random forest model performed best on ER13, achieving the highest accuracy (95.47%) and consistently high AUC, suggesting the strongest model performance on this dataset. On the other hand, all models performed relatively poorly on ER15, with the random forest showing a notably lower recall (as low as 0.729), indicating that this dataset or its samples may be more difficult to classify. This may be related to sample size: ER13 had the largest number of samples, while ER15 had the fewest, suggesting that larger datasets may lead to better model performance.

#### 2.4.4 Feature importance analysis

**Figure 5:**
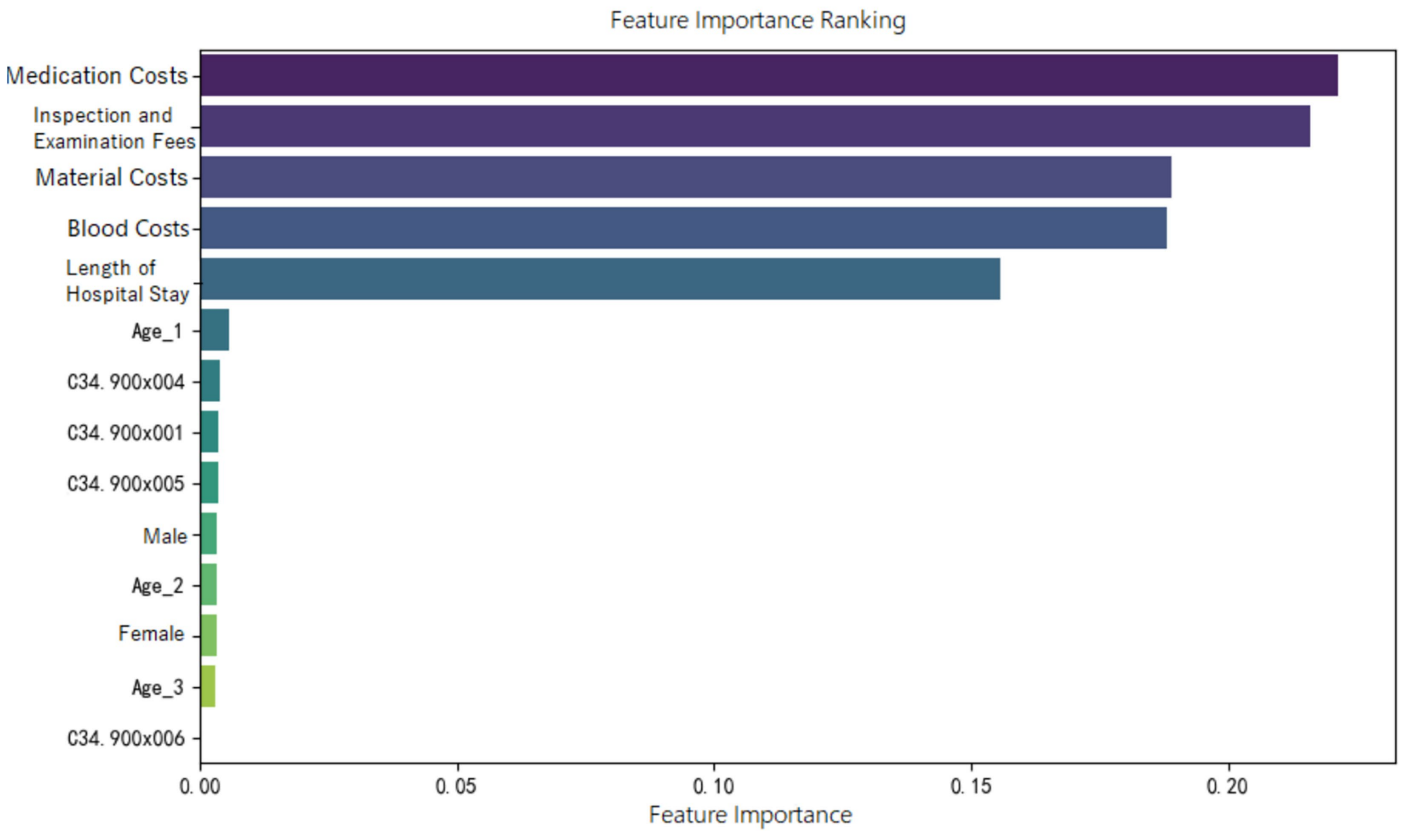
Feature importance ranking of the random forest prediction model in the ER11 group.

**Figure 6:**
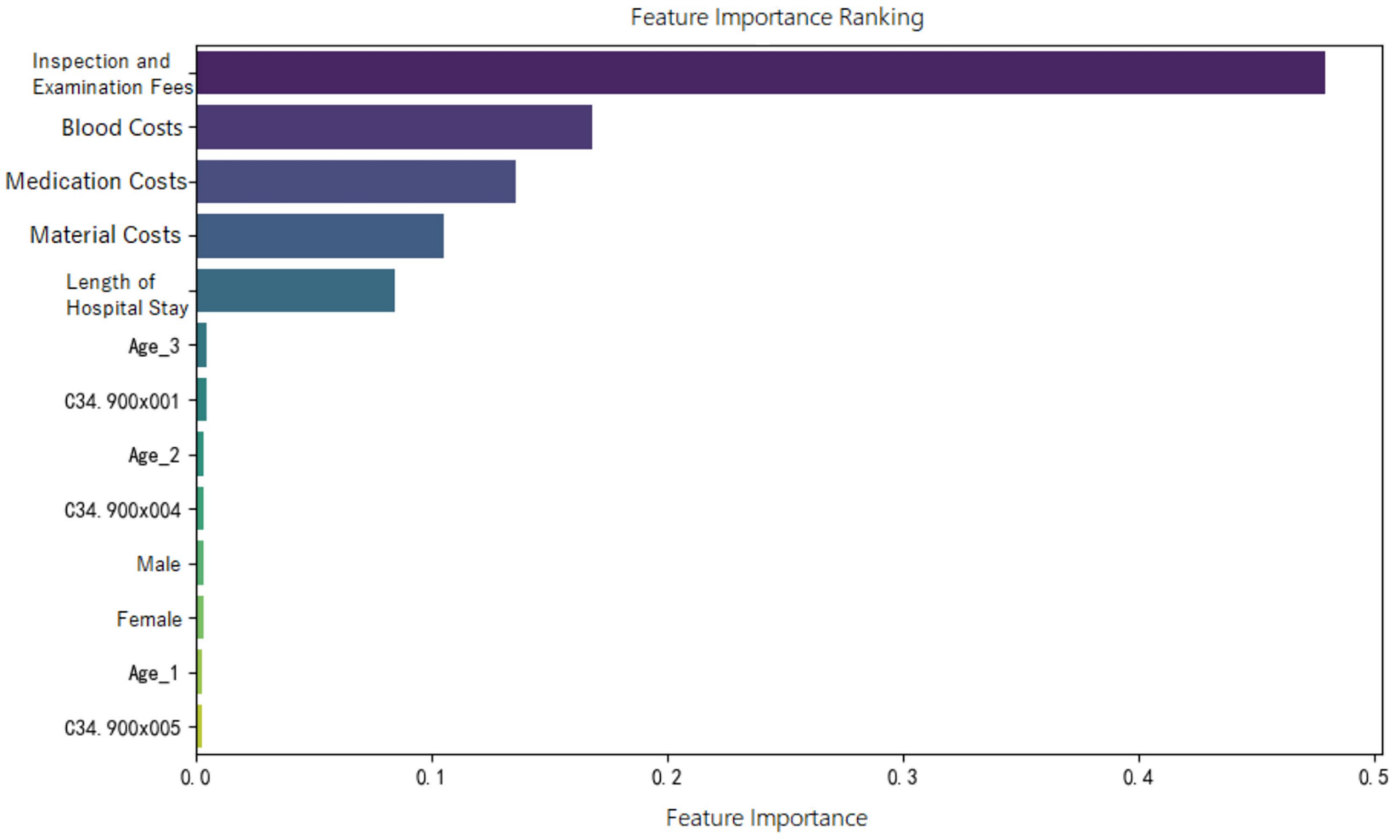
Feature importance ranking of the random forest prediction model in the ER13 group.

**Figure 7:**
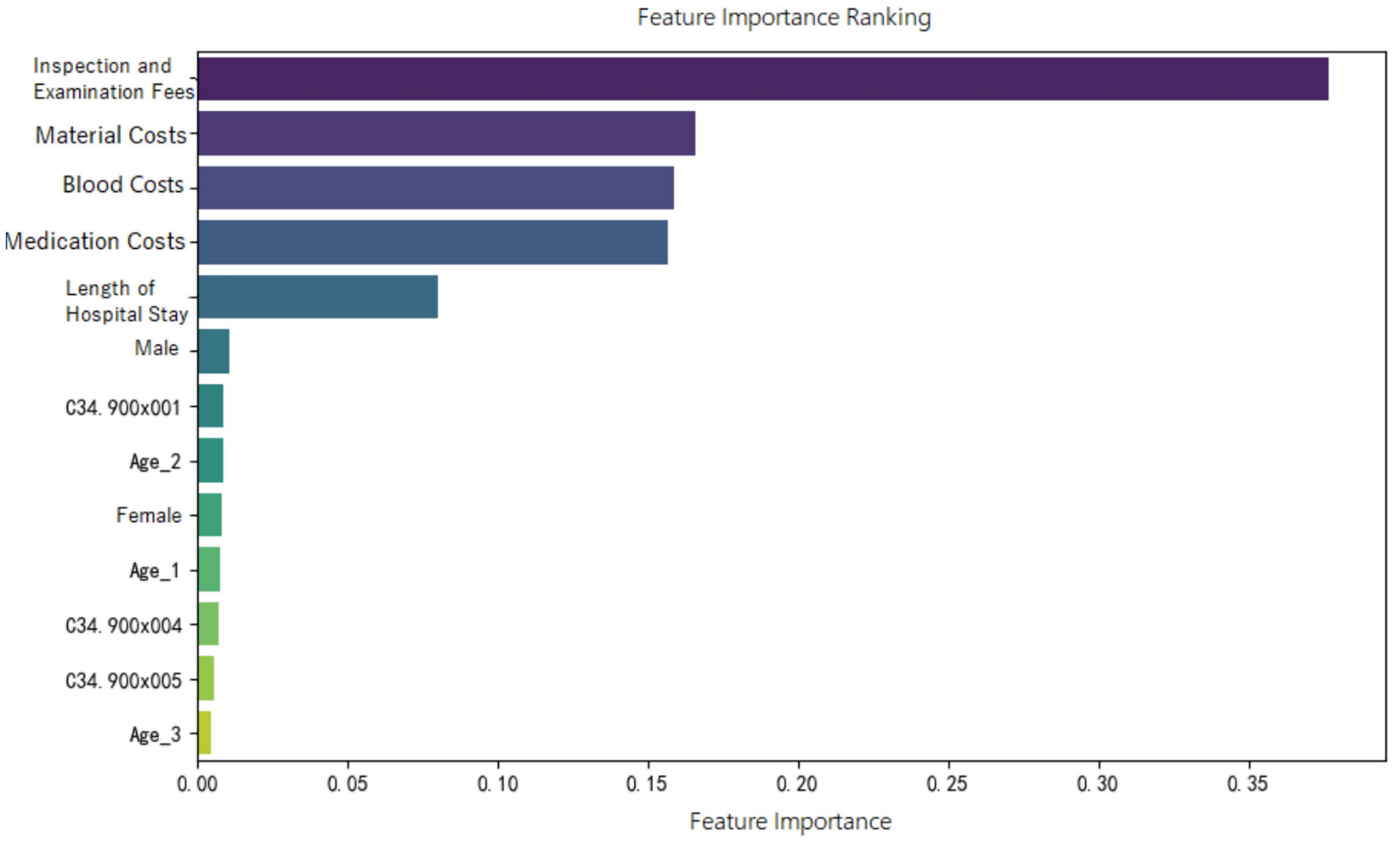
Feature importance ranking of the random forest prediction model in the ER15 group.

Feature importance ranking of various indicators was organized for the random forest model constructed in the three groups, as shown in Figures 5–7. It can be observed firstly, variables such as medication costs, material costs, blood costs, inspection and examination fees, and length of hospital stay contributed significantly to the construction of each model; Secondly, age, gender, and the main diagnostic and coding features of ICD contributed less to the development of the algorithm models.

## 3 Discussion

DRG is a valuable tool for reducing healthcare costs and improving hospital quality and efficiency. The system is technically and administratively complex, and its actual performance depends on organizational behavior. Successful implementation of the DRG/case-mix system requires continuous evidence-based evaluation and monitoring of healthcare services (36). Since the implementation of DRG systems, many countries have reported the phenomenon of discretionary DRG coding by hospitals. A study in Indonesia suggested that narrowing the price differences between DRG groups may help reduce such discretionary coding to some extent (10). In the United States, research showed that the estimated effects of upcoding are not only statistically significant but also economically substantial. Using the most conservative estimates of upcoding, a 3% markup was observed as a result of the MS-DRG system. In 2008, hospital healthcare expenditures in the U. S. totaled approximately $730 billion. Based on the 3% lower-bound estimate, this implies that around $20 billion in excess payments could be attributed to upcoding (13).

Among the many DRG disease groups, this study selected advanced primary lung cancer receiving internal medicine treatments as the research focus because treatment approaches—such as targeted and immunotherapy—are evolving rapidly and lack standardized protocols. This creates challenges for DRG-based payment systems in effectively regulating non-standard medical practices. As previously mentioned, these discretionary and non-standard medical practices are likely to result in low multiplier DRGs. This study aimed to develop machine learning models capable of automatically predicting whether a given DRG group falls under low multiplier cases, thereby laying the groundwork for pre- and mid-process supervision of DRG-based payment during hospitalization. Currently, very few studies have been found that use machine learning to predict or automatically classify DRG multiplicity. A study from Switzerland, similar to ours, applied Random Forest and LASSO-regularized logistic regression to identify variables that predict high-profit and/or high-deficit DRGs. The researchers found that oncological cases were well-funded under the 2012 Swiss DRG system. In particular, a high PCCL (Patient Clinical Complexity Level) score often resulted in classification into a more highly remunerated DRG. As a result, variables such as leukemia and the PCCL score were identified as important predictors of high-profit cases (37).

In this study, descriptive statistical analyses indicated that the demographic distribution (gender, age) of the three selected sample groups aligned closely with the population of advanced lung cancer patients, suggesting that the samples were representative. Furthermore, the Kruskal-Wallis H test showed significant differences in cost-related variables and LoS between the three ER groups. This indicates that version 1.0 of the CHS-DRG classification — particularly its categorization of diagnostic terms and the severity of associated complications — is reasonably designed for advanced lung cancer cases, providing a solid foundation for developing machine learning prediction its models. Additionally, the proportion of high multiplier DRG cases was relatively low across all three groups, suggesting that low multiplier DRG cases are more prevalent. Supporting this, literature reported that low multiplier cases are predominantly found in categories like neurological diseases (MDCB) and respiratory diseases (MDCE), with annual growth rates of 8.14 and 26.15%, respectively (12).

Besides, based on the values of IQR/Median, within each ER group, the distributions of medication, material, blood costs are relatively dispersed, indicating considerable variability among patients. In contrast, inspection and examination fees exhibited more centralized distributions, reflecting higher uniformity and standardization within these groups. This suggests that, within each ER group, medication, material and blood costs are key variables for monitoring potentially inappropriate medical practices.

Moreover, the feature importance of predictive models revealed that variables reflecting “resource consumption”—such as medication costs, material costs, blood costs, inspection and examination fees, and LoS—significantly contribute to the construction of model across all three ER groups. It suggests that these variables have a substantial impact on DRG multiplier payment. Following these, count variables like age, gender, and ICD primary diagnosis codes play a secondary or minor role.

It is also important to emphasize that across all three ER groups, inspection and examination fees consistently show a high level of importance in contributing to the “low multiplier cases,” which may be related to upcoding practices. According to version 1.0 of the CHS-DRG system, in addition to classification under ER1, patients with advanced lung cancer receiving internal medicine treatments may also be grouped under RE1 (malignant proliferative diseases treated with chemotherapy and/or targeted or biological therapies) or RU2 (malignant proliferative diseases treated with immunotherapy). Based on data from the hospital we observed, the RW (Relative Weight) values for ER11, RE11, and RU21 (with the second digit “1” indicating the presence of major complications or comorbidities) are 1.24, 0.78, and 0.72, respectively. The highest RW for ER11 suggests that this group receives the most intensive treatment and incurs the highest level of costs compared to the other two. In contrast, patients classified under RE1 and RU2 are often readmitted for short-term chemotherapy or immunotherapy, and therefore do not require comprehensive re-examinations during hospitalization. If cases that should have been assigned to RE1 or RU2 are instead placed under ER1 due to upcoding motives, they may undergo fewer inspection and examination fees than truly appropriate ER1 cases. This reduction in examinations could lead to lower multiplier DRGs. In this study, we also interviewed several physicians at the hospital. Some admitted that, when filling out diagnostic information, there is a possibility of deliberately assigning patients to more “complicated” DRG groups. This is similar to findings from a Norwegian study, which described such behavior as “a deliberate and systematic shift in a hospital’s reported case mix in order to improve reimbursement (9).”

Additionally, in the ER11 group, which includes cases with severe complications or comorbidities, medication costs have the greatest impact on lower multiplier DRGs. Thus, when DRG classification is accurate, doctors or hospitals, in order to retain a surplus from medical insurance payments, may shift part of the medication treatment to out-of-pocket services, resulting in lower multiplier DRGs.

Finally, the observed differences in the performance of the four machine learning models can be largely attributed to their inherent algorithmic characteristics. The random forest model consistently outperformed the others across the ER11, ER13, and ER15 groups in terms of AUC, accuracy, and precision, particularly excelling in the ER13 dataset where the sample size was the largest. This is likely due to its ensemble structure and robustness against overfitting, as well as its ability to handle complex feature interactions and non-linear relationships. However, its recall exhibited noticeable variability, indicating some instability in identifying low-multiplicity cases under different data splits. In contrast, logistic regression and support vector machine (SVM) models achieved higher recall, making them more suitable for scenarios where minimizing missed diagnoses is a priority, such as pre-claim review or clinical warning systems. Their performance reflects their tendency to classify more instances as positive, thus improving coverage at the cost of lower precision. On the other hand, Naive Bayes models demonstrated the weakest overall performance, particularly in terms of precision and robustness. This is largely due to their underlying assumption of feature independence, which often does not hold in complex healthcare datasets. As a result, their predictive power can be limited in certain clinical contexts, highlighting the need for feature engineering or hybrid modeling approaches to enhance accuracy. Additionally, the models performed best on the ER13 group and worst on ER15, which may be explained by the differences in sample size. ER13 had the largest number of cases, allowing better generalization and smoother ROC curves, whereas ER15 had the fewest samples, leading to lower recall and more volatile performance.

## 4 Conclusion

Firstly, regarding differences in treatment costs and length of stay (LoS), the DRG classification of advanced primary lung cancer patients receiving internal medicine treatment under ER1—based on the severity of comorbidities or complications—appears relatively reasonable and can serve as a basis for comparing DRG-based payment systems across groups.

In addition, based on the findings of this study, during hospitalization for the three ER1 subgroups, particular attention should be paid to whether there is a noticeable reduction in inspection and examination fees, which may indicate upcoding. Other, in the ER11 group, where patients have severe complications, medication treatment should also be closely monitored to prevent intentional underuse of reimbursable drugs.

Lastly, in models for low multiplier DRG prediction, the results suggest that larger datasets tend to enhance model stability and performance. Moreover, model selection should be purpose-driven: Random Forest is preferable for high precision and robustness, while logistic regression or SVM is more suitable when high recall is required. By applying machine learning models to automatically predict low multiplier DRG cases, potential signs of inappropriate medical behaviors can be identified, enabling early intervention and supporting the transition from retrospective reimbursement to a DRG-based prospective payment system.

## Data Availability

The raw data supporting the conclusions of this article will be made available by the authors, without undue reservation.
